# Orthodontic Treatment of Palatally Impacted Canines in Severe Non-Syndromic Oligodontia with the Use of Mini-Implants: A Case Report

**DOI:** 10.3390/medicina59112032

**Published:** 2023-11-17

**Authors:** Marcin Stasiak, Aleksandra Kołodziejska, Bogna Racka-Pilszak

**Affiliations:** 1 Division of Orthodontics, Faculty of Medicine, Medical University of Gdańsk, Aleja Zwycięstwa 42c, 80-210 Gdańsk, Poland; 2 University Dental Center of Medical University of Gdańsk, Dębowa 1a Street, 80-204 Gdańsk, Poland

**Keywords:** cone beam computed tomography, impacted canine, tooth impaction, oligodontia, temporary anchorage device

## Abstract

*Background*: The risk of palatally displaced canines (PDCs) rises in patients with tooth agenesis. The orthodontic extrusion and alignment of PDCs require adequate anchorage to enable tooth movement and control the side effects. There is no paper presenting treatment in the case of severe oligodontia with simultaneous PDCs and the use of mini-implants (MIs) for their orthodontic extrusion. *Case presentation*: A 15-year-old patient presented with non-syndromic oligodontia and bilateral PDCs. Cone beam computed tomography revealed that both PDCs were in proximity to the upper incisors’ roots. There was no evident external root resorption of the incisors. The “canines first” approach was chosen. MIs were used both as direct and indirect anchorage. First, the extrusive forces of cantilevers were directed both occlusally and distally. Next, the buccal directions of forces were implemented. Finally, fixed appliances were used. PDCs were extruded, aligned, and torqued. Proper alignment and occlusion were achieved to enable further prosthodontic restorations. *Conclusions*: The use of MIs made it possible to avoid collateral effects, reduce the risk of complications, and treat the patient effectively. MIs provide adequate anchorage in demanding cases. The use of MIs for the extrusion of PDCs made it possible to offer this treatment option to patients with severe oligodontia. The presented protocol was effective and served to circumvent treatment limitations associated with an inadequate amount of dental anchorage and a high risk of root resorption.

## 1. Introduction

Hereditary tooth agenesis could be classified as hypodontia, oligodontia, and anodontia. Hypodontia is a mild form, which involves a lack of several teeth except the third molars. Oligodontia is a state where six or more teeth, excluding the third molars, are missing. Anodontia is associated with a complete lack of teeth.

The prevalence of non-syndromic hypodontia ranges from 3% to 10%, whereas the more severe oligodontia shows a prevalence of 0.1–0.5%. Anodontia is found almost exclusively in syndromic cases [1]. Oligodontia can present not only as an isolated condition but can also present as a part of syndromes [2,3,4]. A genetic factor is commonly described as a cause of isolated oligodontia. Mutations in the EDAR, EDA, PAX9, MSX, WNT10A, and LRP6 genes are associated with non-syndromic oligodontia [5,6,7]. Moreover, environmental factors and host factors such as radiotherapy, chemotherapy, disease/infection, viral infection during pregnancy, and metabolic imbalances could lead to germ-formation disturbances [8].

Impaction of the maxillary permanent canines is the second most common form of tooth impaction after the third molars. Impacted maxillary canines are often divided into two groups—palatal and vestibular impaction cases. The impacted canine is located on the palatal side of the maxilla in 85% of cases, whereas vestibular localization is present in 15% of cases [9]. The prevalence is highly associated with ethnicity. Palatally displaced canines (PDCs) are more frequent in the Caucasian population, while buccally displaced canines are more common in the Asian population [10,11]. There is no single cause of PDCs. The etiology is associated with local hard tissue obstruction, local pathology, disturbance of the normal development of the incisors, hereditary or genetic factors, and tooth agenesis [12]. The risk of maxillary canine impaction rises in patients with non-syndromic tooth agenesis due to a lack of root guidance [13].

Management of PDCs is one of the most challenging orthodontic treatments to conduct. The interceptive approach with primary canine extraction could have a favorable effect on PDCs. However, this attempt is still controversial, showing a correction that was not significantly greater than in untreated controls [14]. Alternative treatment should be performed if no radiographical correction of PDC is detected after 12 months from primary canine extraction. There are different treatment alternatives, which should be individually considered, including observation, orthodontic traction after surgical exposure, autotransplantation, and tooth extraction. 

Observation may be considered when the patient rejects orthodontic treatment, surgery presents a high risk, and there are no cysts and no contact with other teeth.

Surgical exposure may be performed with the closed or open technique. The closed technique involves surgical uncovering of the canine with a full-thickness mucoperiosteal flap dissected off the bone. The bone that covers the canine is removed, and an attachment with a chain or metal ligature is bonded to the exposed tooth. Subsequently, the palatal flap is repositioned and sutured back in place. The chain or ligature penetrates through the soft tissue and provides future orthodontic traction. In the open technique, the surgical uncovering is followed by the removal of mucoperiosteal tissue from around the tooth. Then, an orthodontic abutment is placed, and the exposed area is covered with a dressing. The teeth are expected to present better periodontal health after the closed exposure. However, the current evidence suggests that neither the open nor the closed surgical technique is superior to any of the outcomes [15]. The success rate of combined surgical and orthodontic treatment of PDCs depends on their position and relation to the anatomic structures and neighboring teeth [16]. The presence of ankylosis affects the outcome of the orthodontic extrusion procedure. It might have afflicted the impacted tooth either a priori or as a result of the earlier surgical or orthodontic maneuvers [17]. Apicotomy might be considered as an additional procedure in the case of dilaceration or apical ankylosis [18]. 

Autotransplantation is an alternative approach, which makes it possible to align the tooth in one procedure. It could be taken into consideration when the position of the impacted tooth is of bad prognosis, and the traction may result in unwanted collateral effects, such as root resorption or the periodontal impairment of the adjacent teeth [19]. However, this attempt may also lead to posttreatment complications. The necrosis or the resorption of the transplanted teeth has been reported. The most crucial factors for the survival of transplants and their continued development are, on average, three-quarters length of root development, a wide open apex, and a careful surgical technique that preserves both the periodontal ligament and the gingival margin [19]. An alternative option is block autotransplantation, where the tooth is repositioned with a transplanted block of surrounding bone. This procedure is performed on teeth with the finished development of the roots and closed apices [20]. 

Finally, the clinician may decide to extract the impacted teeth when there is no possibility of orthodontic extrusion, the patient rejects orthodontic treatment, and contraindications for observation are present.

The orthodontic extrusion and alignment of PDCs require adequate anchorage to enable orthodontic tooth movement and to control side effects. Teeth segments, which are stabilized with stiff stainless-steel orthodontic archwire, commonly serve as an anchorage. These segments could be further reinforced with conventional anchorage appliances such as a transpalatal arch or a Nance plate. The problem arises when there are no available teeth that meet the anchorage requirements, or their condition precludes their usage. Currently, temporary anchorage devices such as orthodontic mini-implants (MIs) serve to circumvent this limitation. MIs may be used both as direct and indirect anchorage [21]. Direct anchorage means that the force is placed directly to the MI, while in indirect anchorage, the MI is bonded with the teeth to avoid unwanted movement due to the reciprocal orthodontic forces.

To the best of the authors’ knowledge, no paper has been published presenting preprosthetic orthodontic treatment in the case of severe oligodontia with simultaneous PDCs and the use of MIs for their orthodontic extrusion. The aim of the study was to present a case report describing the orthodontic treatment of the patient with PDCs in severe oligodontia. The treatment was performed with the use of MIs as direct and indirect anchorage and fixed appliances. The current follow-up with retention is one year.

## 2. Materials and Methods

The study was conducted in accordance with the Declaration of Helsinki and approved by the Ethics Committee of Medical University of Gdańsk (protocol code: NKBBN/96/2023, date of approval: 24 February 2023). Written informed consent was obtained from the patient to publish this paper. The study was performed according to the CARE (for Case Reports) guidelines [22].

A 15-year-old patient presented on 29 March 2017 to the Division of Orthodontics to conduct an orthodontic treatment. The patient was referred by an orthodontist from private practice, and no orthodontic treatment was previously performed. Their main concern was the smile esthetic, and they were in good health and reported no other health issues. 

**Figure 1:**
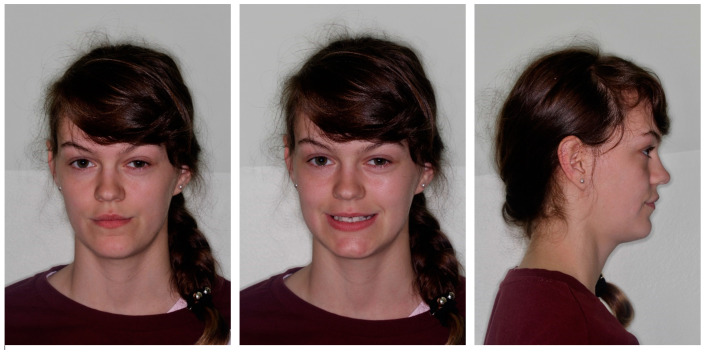
Initial extraoral photos.

The profile was straight, the lips were retrusive to the E line, and the nasolabial angle was a bit decreased. The tooth display was reduced, and the patient showed below 75% height of the central incisors. The smile arch was flattened. Moreover, buccal corridors were present due to the lack of posterior teeth (Figure 1).

**Figure 2:**
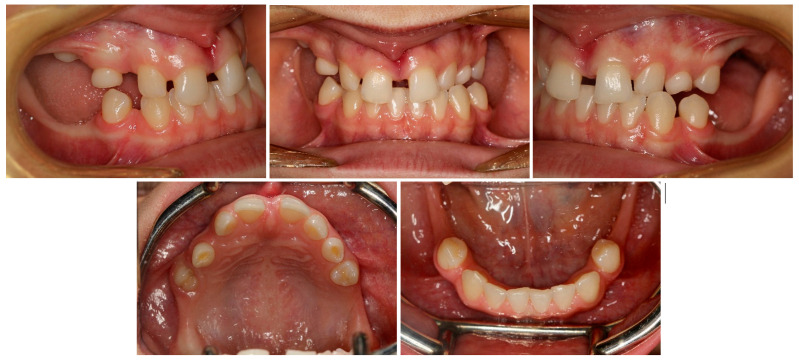
Initial intraoral photos.

**Figure 3:**
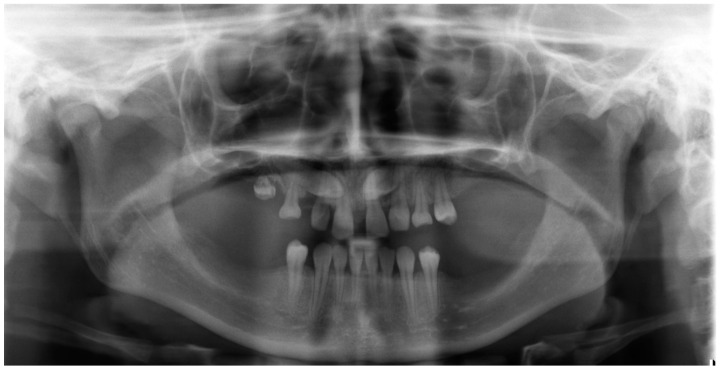
Pretreatment panoramic radiograph.

**Figure 4:**
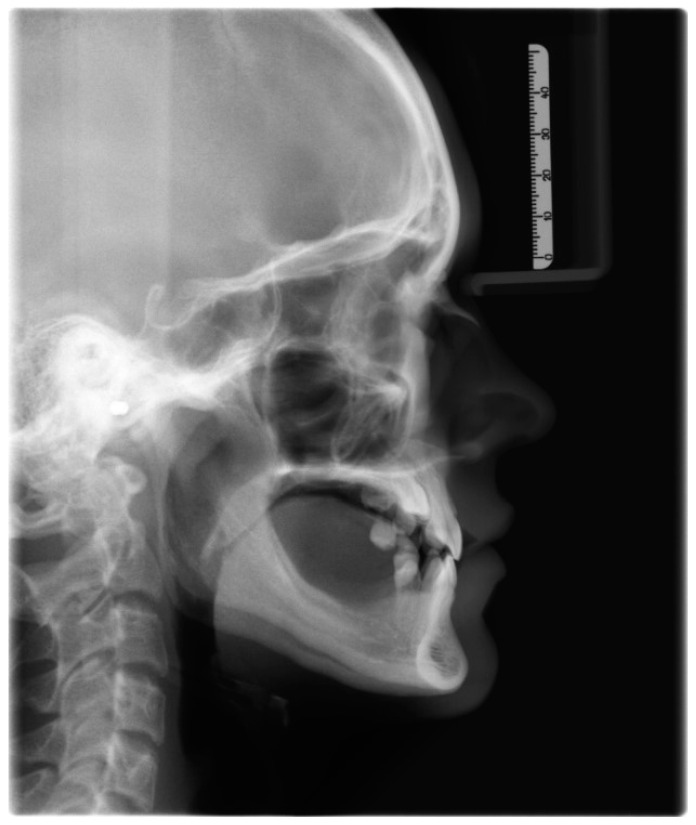
Pretreatment lateral cephalogram.

**Figure 5:**
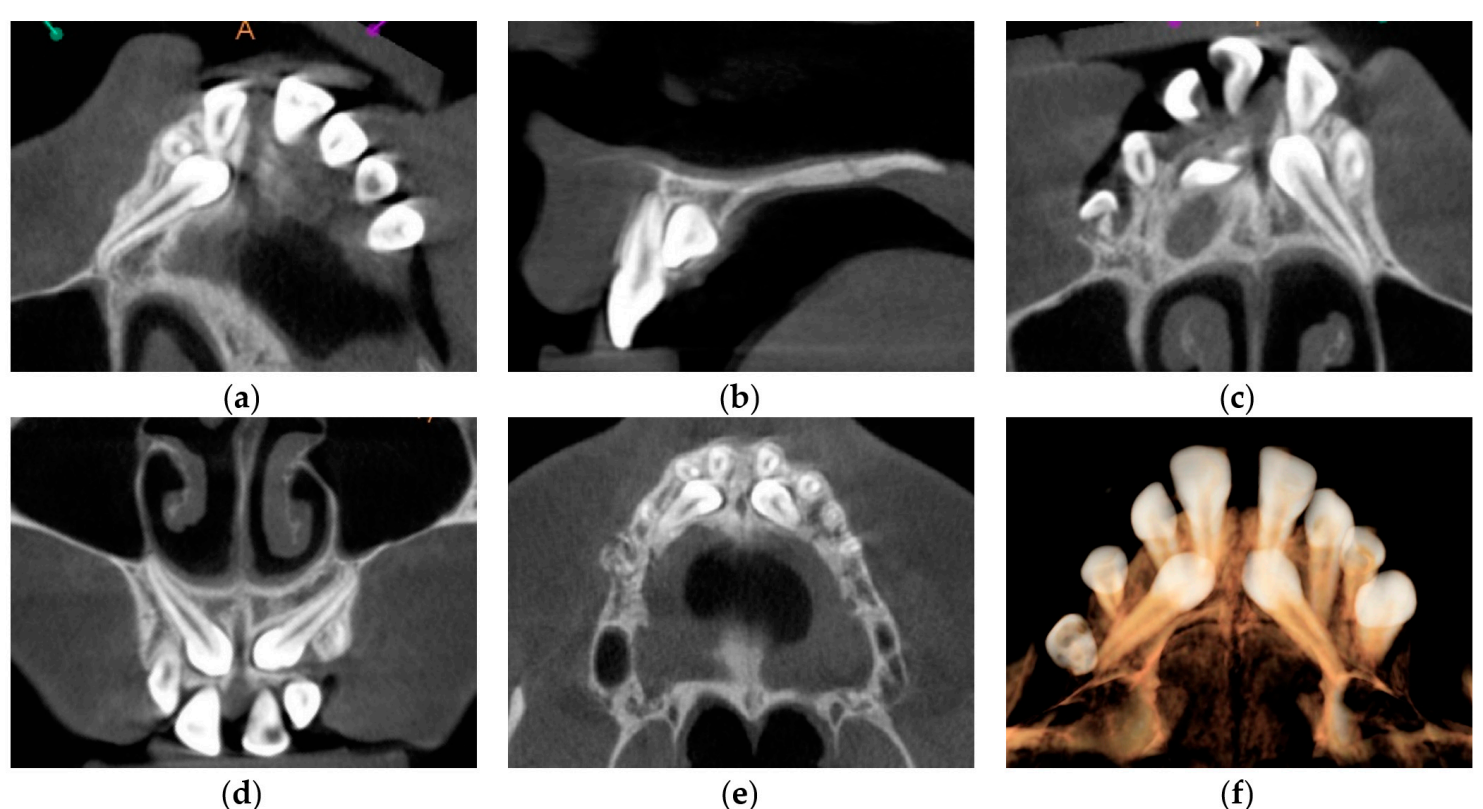
Cone beam computed tomography cross-sections with palatally impacted maxillary canines. (**a**) Upper right canine; (**b**) Proximity of the upper left canine and the upper left central incisor; (**c**) Upper left canine; (**d**) Transversal cross-section; (**e**) Horizontal cross-section; (**f**) Cone beam computed tomography rendering.

The permanent incisors, first left permanent premolar, deciduous canines, and right deciduous second molar, were present in the upper arch. The deciduous molar presented moderate reinclusion due to the ankylosis. The teeth from the first right to the first left permanent premolars were present in the lower arch. Spacing between the teeth was both in the upper, including diastema, and in the lower arch. The midlines did not correlate. The upper midline correlated with the midline of the face. The lower midline was shifted to the left, together with a deviation of the Pogonion point. There was left side crossbite on teeth no. 22, 63, and 24 (Figure 2). Orthodontic examination revealed non-syndromic oligodontia with no family history and bilateral palatal impaction of the maxillary canines (Figure 3). Cephalometric analysis revealed slight class III malocclusion with retrusion of the lower incisors (Figure 4 and Table 1). The analysis was perfomerd using Ortodoncja 9 software (version 9.2.2, Wrocław, Poland). Cone beam computed tomography (CBCT) revealed that both PDCs were in proximity to the upper incisor’s roots. Tooth no. 13 presented 2.1 mm of root dilaceration. There was no evident external root resorption of the incisors. The PDCs were close to the oral cavity. Bone deficiency of the alveolar processes was present in the location of the missing teeth (Figure 5).

The treatment objectives were defined after clinical examination, full orthodontic diagnostic records analysis, and consultation with the oral surgeon. No orthognathic surgery was planned due to the acceptable facial appearance and lack of patient demand. PDCs were qualified for surgical exposure and subsequent orthodontic traction. The proximity of canines’ crowns and incisors’ roots affected the planning of the orthodontic mechanics, which aimed to avoid unfavorable root resorption. The traction forces were planned to be directed both occlusally and distally in the initial treatment period. In the next phase, the buccal force directions were planned to be implemented. Finally, adequate torque and final position were planned. The upper and lower arch should be aligned, the spaces closed, and the midlines correlated. Prosthetic restorations, both on the patient’s teeth and on the dental implants, were planned to be performed after the finishing of the orthodontic treatment. 

The possible treatment alternatives were observation, extraction, and tooth autotransplantation. Those options were abandoned. Observation was rejected due to the contact of the canines with the central incisors’s roots and the risk of their resorption. Roots of the canines were formed more than three-quarters of their length, which worsened the prognosis of a successful autotransplantation. Extraction was also rejected due to the oligodontia, the possibility of successful orthodontic treatment, and the prosthetic substitution needed in the esthetic area in the case of these extractions. The patient and their parents also rejected those options. Informed consent for treatment was obtained.

## 3. Results

### 3.1. Treatment Process

**Figure 6:**
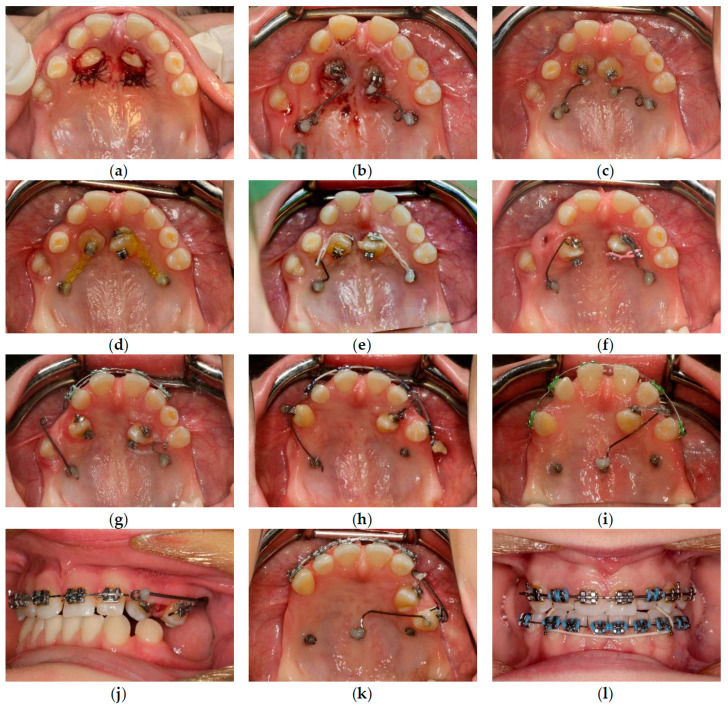
Treatment process. (**a**) Surgical exposure; (**b**) Placement of palatal alveolar mini-implants and installation of cantilevers; (**c**) Orthodontic extrusion with cantilevers in distal and downward direction; (**d**) Orthodontic extrusion with power chains; (**e**) Derotation of canines with power chains; (**f**) Extraction of upper right deciduous canine and buccal movements of impacted canines; (**g**) Palatal alveolar mini-implants and cantilevers for buccal tooth movements; (**h**) Buccal alveolar mini-implant and cantilever for buccal movement of upper left canine; (**i**) Mini-implant in the palatal suture used as direct anchorage for upper left canine and bend-out for upper right canine; (**j**) Cantilever for buccal movement of upper left canine; (**k**) Mini-implant in the palatal suture used as indirect anchorage; (**l**) Intermaxillary elastics from palatal buttons on upper lateral incisors to the lower arch.

**Figure 7:**
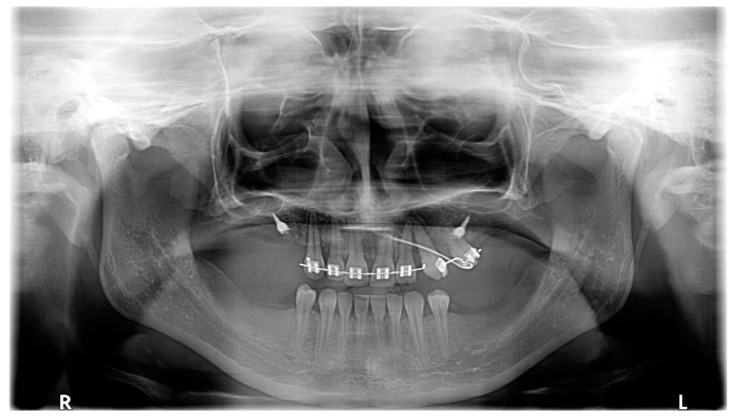
Panoramic radiograph performed after orthodontic extrusion.

**Figure 8:**
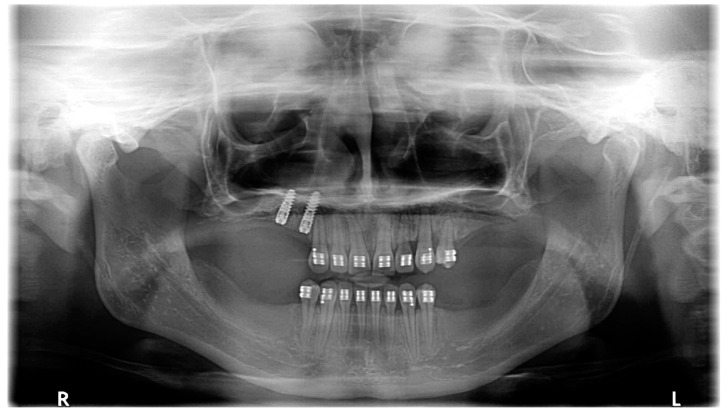
Panoramic radiograph performed during the finishing phase.

**Figure 9:**
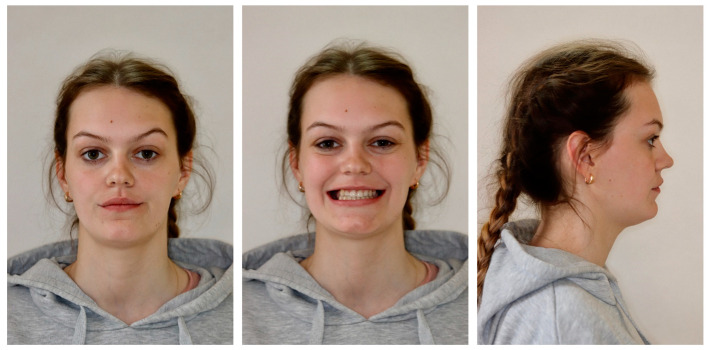
Final extraoral photos.

**Figure 10:**
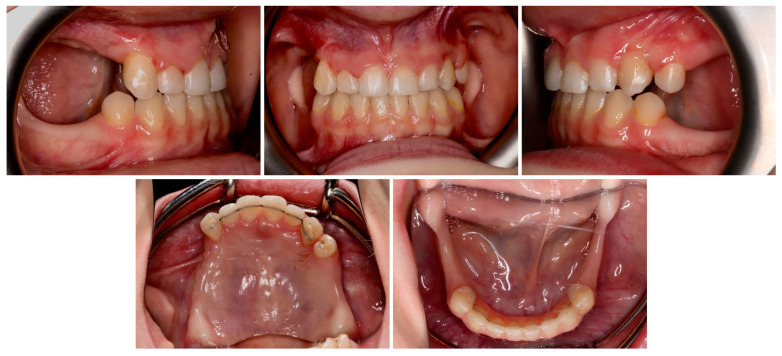
Final intraoral photos.

**Figure 11:**
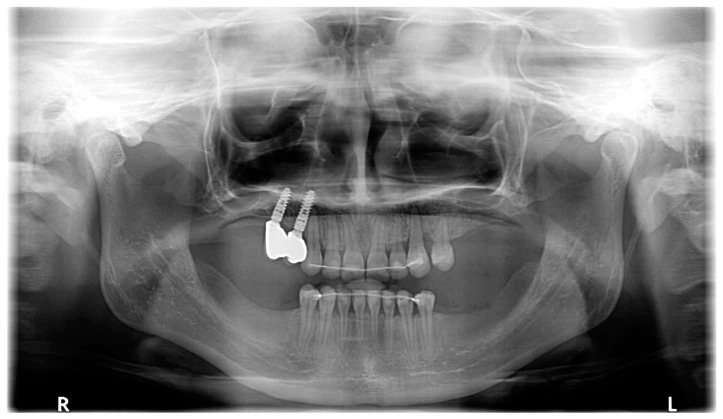
Posttreatment panoramic radiograph.

**Figure 12:**
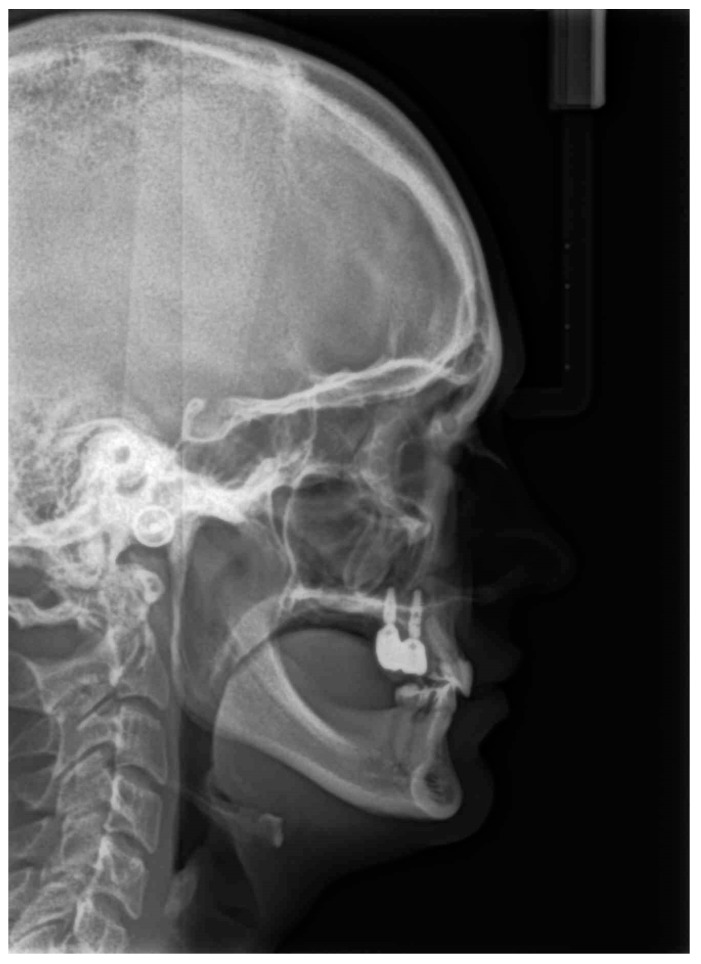
Posttreatment lateral cephalogram.

**Figure 13:**
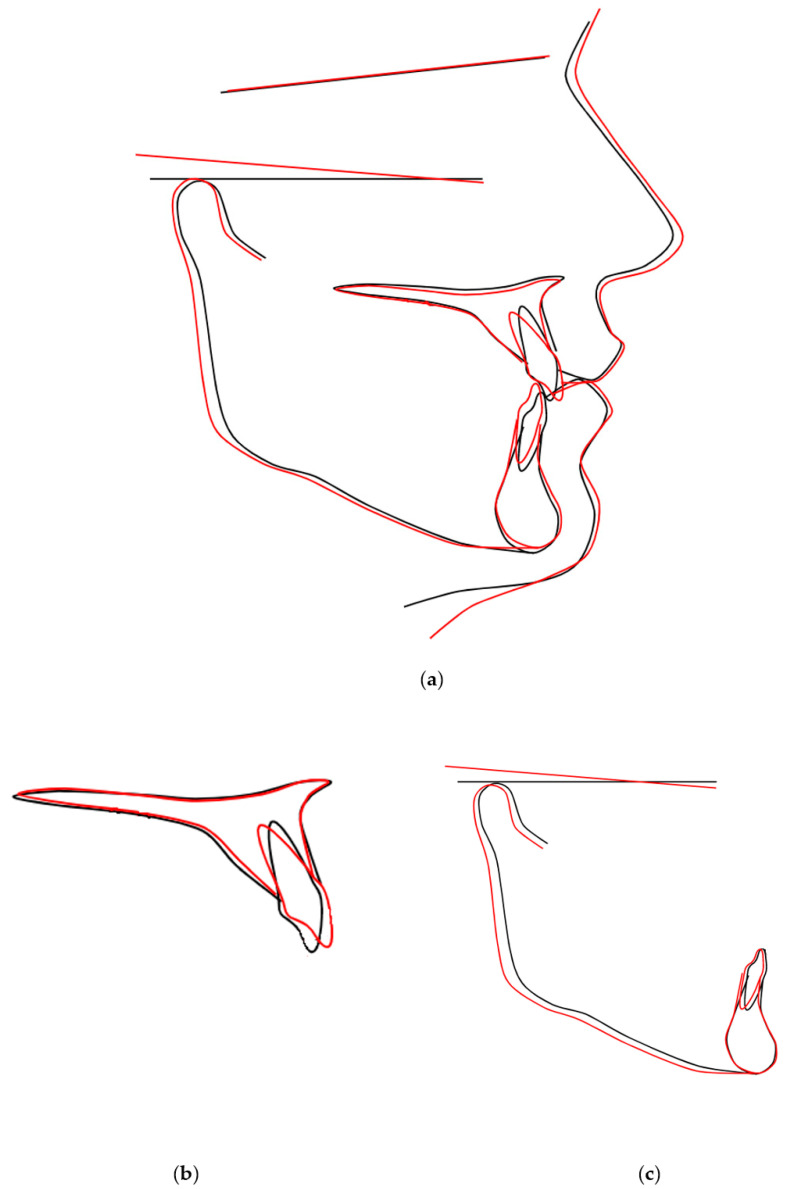
Superimposition of the initial (black color) and final (red color) cephalometric radiographs to monitor skeletal and soft tissue changes. (**a**) Superimposition of cranial base structures; (**b**) Maxillary superimposition; (**c**) Mandibular superimposition.

Open flap surgery was chosen by consensus based on the preferences of both the orthodontist and oral surgeon due to the proximity of the PDCs to the oral cavity. The active phase of orthodontic treatment took 4 years and 7 months (13 October 2017–16 May 2022). This phase could be divided into three parts: the first stage, including surgery, MIs, cantilevers, and elastics (8 months, 13 October 2017–14 June 2018); the second stage, including fixed upper appliance and MIs (2 years and 9 months, 14 June 2018–17 March 2021); and the third stage, including treatment with fixed upper and lower appliances (1 year and 2 months, 17 March 2021–16 May 2022). The whole treatment period with the fixed upper appliance was 3 years and 11 months (14 June 2018–16 May 2022). MIs were manufactured from grade 5 titanium implant material (TiAl6V4). Insertion sites for MIs were carefully planned based on the CBCT examination. Control visits were conducted every 2–3 weeks during the first phase of the orthodontic treatment. This was due both to the rapid acceleration phenomenon after the surgery [23] and the lack of a fail-safe mechanism during the force application with cantilevers. The impacted canines were moved as planned—first occlusally and distally, then buccally (Figure 6). For esthetic and psychosocial reasons, the upper deciduous canines were extracted just before the fixed upper appliance placement (Figure 7). The main aims of the treatment with fixed upper appliance were crossbite correction with asymmetric V-bends and obtaining adequate torque for the canines with their root palpability in the vestibule. A lower appliance was used to obtain better overjet and intercuspation by using elastics (Table 2, Figure 6). The main goals of the presented treatment—extrusion of PDCs and achieving both alignment and occlusion, enabling further prosthodontic restoration—were obtained (Figure 8). After the active phase of the orthodontic treatment, the upper and lower fixed retention wires were placed. An upper thermoformable retention splint was also used. Final clinical records show the result of the active orthodontic treatment (Figure 9 and Figure 10). Final radiographic records were obtained (Figure 11 and Figure 12). Cephalometric analysis was perfomerd using Ortodoncja 9 software (version 9.2.2, Wrocław, Poland). Superimposition of the initial and final cephalometric radiographs was performed using WebCeph™ (version 1.5.0, AssembleCircle Corp., Seongnam, Republic of Korea). Next, the treatment changes were assessed (Table 3 and Figure 13), the analysis of which showed proclination of both the upper and the lower incisors. At the same time, the horizontal growth direction and the anterior rotation of the mandible worsened the class III skeletal relationship. 

### 3.2. Follow-Up

**Figure 14:**
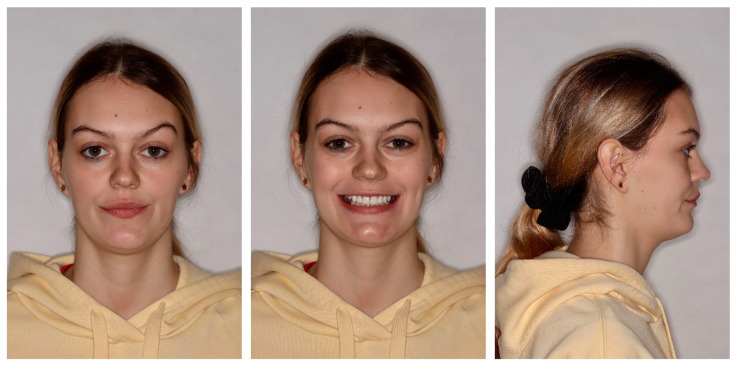
Extraoral photos after the initial phase of prosthetic treatment.

**Figure 15:**
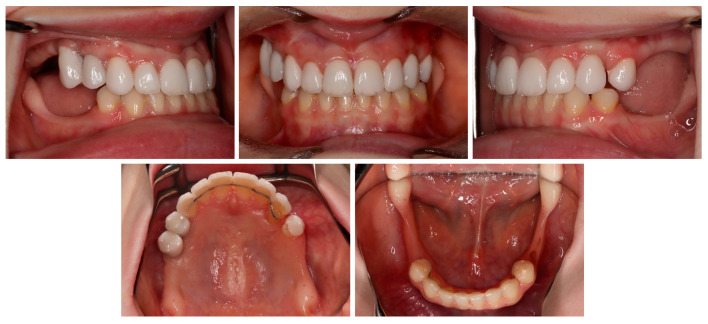
Intraoral photos after the initial phase of prosthetic treatment.

Figure 14 and Figure 15 present the result after the initial phase of prosthodontic treatment with good esthetics and functional recovery.

**Figure 16:**
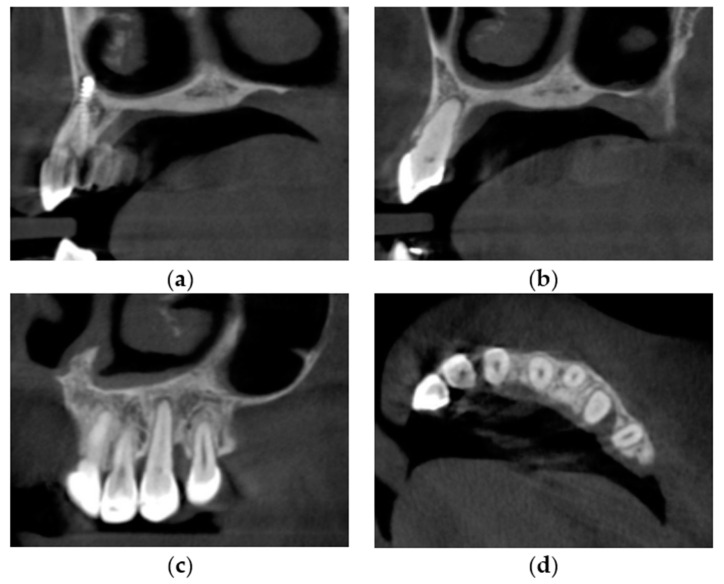
Changes in the canines’ anatomy due to conducted orthodontic treatment. (**a**) Upper right canine; (**b**) Upper left canine; (**c**) Bone defect of upper left canine—sagittal cross-section; (**d**) Bone defect of upper left canine—horizontal cross-section.

The CBCT was performed after the treatment to assess the further implantation possibilities. It also made it possible to estimate possible complications in terms of root resorption and alveolar bone defects. Levander et al. [24] categorized four levels of apical root resorption. Stage 1 describes irregular root contour; stage 2 is less than 2 mm of apical root resorption; stage 3 is apical root resorption, amounting to between 2 mm and one-third of the original root length, and stage 4 is root resorption, exceeding one-third of the original root length. Every tooth, except no. 13 and 23, could be classified as level 2. Teeth no. 13 and 23 showed stage 3 with 2.6 mm and 2.2 mm of apical root resorption, respectively. Moreover, the primary dilaceration of tooth no. 13 was completely resorbed during the orthodontic treatment. However, metal artifacts due to the presence of the dental implant and prosthetic restoration made the assessment difficult (Figure 16). The lengths of the roots before and after the treatment are presented in Table 4.

There was no significant mobility of tooth no. 24 after 8 months of follow-up. 

The periodontal examination took place during the follow-up visit at 8 months. The examination was conducted according to the British Society of Periodontology guidelines for basic periodontal examination (BPE) [25]. Only two anterior sextants were taken into consideration due to the oligodontia. The score of the sextants was 1. The probing depth was below 3.5 mm, but bleeding points qualified the sextants to group 1. The probing depth was examined at four points (mesial, distal, palatal, and labial/buccal). Every tooth, except no. 23, had a probing depth under or equal to 2 mm. The probing depth was 3 mm in the distal point of tooth no. 23. The palatal probing depth of tooth no. 23 was 1 mm. Concomitantly, there was a gingival recession of 2 mm on the palatal distal side. Finally, 3 mm of clinical attachment loss (CAL) on the palatal site was present. These clinical measurements were compared with the posttreatment CBCT analysis. There was a two-wall bone defect of tooth no. 23 with the presence of labial alveolar bone. The distance from the cementoenamel junction to the alveolar ridge was 1.5 mm, and the critical amount of 2 mm for dehiscence on the CBCT [26] was not fulfilled. The depth of the defect from the alveolar ridge was 3 mm. Therefore, a supra-alveolar attachment of 1.5 mm was present.

The alveolar bone condition of teeth no. 21 and 22 improved during the treatment. There were initially cortical bone fenestrations in the region of the root’s apices. After the treatment, cortical bone was present on the labial surfaces of the apices. Positive torque prescription and full-width wires resulted in palatal torque of the roots.

The CBCT examination also revealed the canal obliteration of tooth no. 23 (Figure 16). There was neither a color change nor a decreased reaction to the cold test during the clinical examination. The tooth was left for observation. In the case of inflammation or discoloration, endodontic or surgical treatment should be performed. Moreover, no evidence of ankylosis was identified during the follow-up examination.

The canines-first approach with the use of MIs made it possible to avoid collateral effects, reduce the risk of complication, and treat the patient effectively. 

The current follow-up period is one year. Bone grafts are planned both in the mandible and in the left quadrant of the maxilla. Next, dental implants will be placed. Following the osseointegration of the implants, fixed prosthetic restorations will be performed to obtain stable occlusion and proper function. These procedures have not yet been performed due to the patient’s financial constraints and the lack of reimbursement from the public payer. Completion of prosthetic rehabilitation will be carried out in the future after the patient has collected funds. 

## 4. Discussion

The reported success rate of impacted canines’ extrusion among adults is 69.5%, compared with 100% among younger patients [27]. The failures resulted mostly from inadequate anchorage (48.6%), mistaken location and directional traction (40.5%), and ankylosis (32.4%) [17].

There is no strong evidence to support the usage of CBCT as a “first line” imaging method for the assessment of PDCs. The current imaging method of choice is still the conventional dental radiography [28]. On the other hand, CBCT provides relevant clinical information (canine position, damage of the adjacent teeth, severity index) with a significant impact on treatment decisions (biomechanics, patient education, treatment time estimation) [16]. Following the panoramic radiograph evaluation, the CBCT of the maxilla region was prescribed to obtain more specific diagnostic information. This is in line with the ALARA (as low as reasonably achievable) principle [29]. The smallest imaging area size compatible with the situation should be selected, aiming to reduce the radiation dose [28]. Therefore, a 10 cm × 5 cm field of view was chosen. The patient was an adolescent, and that type of radiological examination was the most beneficial according to the effective dose and its clinically significant diagnostic value. 

The potential complications during the treatment were the resorption of the adjacent teeth, ankylosis, loss of the attachments on the canines, and collateral effects on the adjacent teeth [17]. There was a high risk of the incisors’ root resorption during the orthodontic treatment. The first cause was the proximity of their roots and the canines’ crowns and the resultant potential collision during the orthodontic tooth movement [17]. That collision might have been caused both by the inadequate extrusion force direction delivered to the impacted teeth and by the incisors’ uncontrolled flaring. Therefore, it should have been taken into consideration when planning the orthodontic mechanics. The extrusion force was first directed distally and occlusally. After the canines fully appeared in the oral cavity, the force could be redirected buccally to slowly align them into the arch. This is also in line with a finite element study [30], which reported that treatment should be initiated with vertical and distal forces. These forces yield significantly lower stress on the PDCs. Uncontrolled flaring is frequently obtained at the beginning of treatment with fixed orthodontic appliances and is characterized by the buccal movement of the crown and concomitant palatal movement of the root. The risk of root resorption was also exacerbated due to the skeletal class 3 camouflage treatment, which is associated with the potential collision of the root apices with the palatal cortical bone [31]. Moreover, tooth no. 13 had root dilaceration before the treatment, which has been reported as a root resorption risk factor [32]. In addition, dilaceration is a potential limitation in orthodontic tooth movement. The presence of the impacted teeth prolongs the treatment, which is a further risk factor [31]. Therefore, the canines-first approach was chosen to provide goal-oriented orthodontic mechanics, to shorten the treatment time with straight wire appliances, and finally to reduce the complication risk. According to this approach, treatment was started with the use of MIs and cantilevers instead of the initial fixed appliance placement. 

Surgical exposure with the open technique was chosen due to the proximity of the canines to the oral cavity and potentially lower levels of resistance to orthodontic traction forces. Moreover, the open technique made the canines’ surfaces visible for the whole treatment time. Therefore, if any loss of the attachment occurred, it could be placed on the next visit without the need for an additional surgical procedure. Concomitantly, no attachment loss was experienced during the treatment.

The use of MIs made it possible not only to avoid collateral effects derived from tooth anchorage but also to treat the patient effectively. The stability of the MIs depends on various factors, including the site of the implantation, the vicinity of the surrounding structures, the thickness of the cortical bone, and the type of MI [33,34]. The cortical bone should be at least 1 mm thick to provide enough retention [35]. The thicker the cortical bone, the better the MI primary retention and the better the long-term stability obtained [36]. Concomitantly, oligodontia results in bone reduction of the alveolar process. Therefore, the potential use of MIs was limited to several locations that offered favorable or acceptable bone conditions. The MI insertion sites were chosen after the careful assessment of CBCT examination in terms of both the bone availability and mechanical requirements for the orthodontic tooth movement. All the MIs were placed in cortical bone with a thickness exceeding 1 mm. Both MIs placed in the palatal alveolar bone presented satisfactory stability, which enabled the use of cantilevers and successful orthodontic tooth movement of the PDCs. MIs placed both in the buccal alveolar bone and in the infrazygomatic crest area were lost. This may have resulted from the reduced bone availability and proximity of mucosa [33]. The last MI was inserted in the palate in the median position after the above-mentioned were lost. That MI was used both as direct and as indirect anchorage. Successful stability of the median MI was then obtained. However, this location seems to be controversial. On the one hand, there is a risk of palate growth impairment due to the median MI insertion [37]. On the other hand, there is a risk of stability loss if the palatal suture is not fully calcified [37].

The patient was 17 years old at the time of the insertion. The suture maturation was assessed as stadium C according to the classification presented by Angelieri et al. [38] at the beginning of treatment. Concomitantly, the midline insertion of the MI was performed 2 years after that initial CBCT examination. In the posttreatment CBCT, stadium D was present. The median location was chosen due to both preferable bone availability in the median location and the lack of collision risk with orthodontic tooth movement, compared to the paramedian one. All three successful palatal MIs showed good primary stability and adequate resistance to reaction forces during the orthodontic tooth movement. This was probably not only due to the better bone availability and its quality but also to the lack of inflammation resulting from soft tissue irritation. 

The MIs were placed directly without the use of any surgical templates. Surgical guides offer the opportunity for more precise and, therefore, potentially more successful MI placement. However, deviation from the insertion location that was planned during the preparation of the surgical guide should also be taken into consideration [39,40].

Temporary anchorage devices play a major role in protecting the adjacent teeth from excessive orthodontic forces. The possible overloading of the anchorage teeth may lead to ischemia and periodontal damage when greater forces are used for more than 6–8 weeks. As a consequence, root resorption could occur [31]. MIs used for direct anchorage disperse the forces acting on the MIs, thus avoiding the harmful effect and undesirable movement of the remaining teeth. After the PDCs were extruded into the oral cavity due to the vertical and distal force vectors, the forces were changed in the buccal direction. The same MI was used both during vertical and buccal movements on the right side due to the lack of the upper first premolar and the resultant lack of collision with an activated cantilever. On the left side, another device was applied due to the collision of the cantilever with the upper first premolar. However, the MIs in both the buccal alveolar region and the infrazygomatic crest were lost. Therefore, the additional MI was placed in the palatal suture and used first as direct and subsequently as indirect anchorage for the buccal movement of the left upper canine. Such indirect anchorage is not a preferable option since it means that the traction of the PDC could generate collateral forces on the teeth connected with the MI [21]. A significant widening of the periodontal space of the upper left premolar was detected on the control panoramic X-ray. The lateral resorption of tooth no. 24 was noticed on the above-mentioned X-ray and on the posttreatment CBCT. This may have been the result of the reactive torsional forces. They could have occurred even though the indirect anchorage unit was rigid. However, studies show that rigid indirect anchorage causes less anchorage loss and lower root resorption risk but may put more stress on the MI. Moreover, it increases the probability of the screw being lost [41]. 

The therapeutic objectives were reduced due to the upper left premolar’s excessive mobility. Therefore, an adequate vertical position and final intercuspation were not achieved. 

The canal obliteration and the periodontal impairment of tooth no. 23 might be caused by excessive continuous forces, which could lead to trauma of the periodontium through a mechanism similar to the luxation traumas visible in adolescents [42,43]. This observation suggests the importance of regional anatomy and controlling the force during orthodontic traction.

Despite the oligodontia, no removable prosthetic appliances were used during the orthodontic treatment. First, the canines were tracked from the palate so there would be a physical obstacle during the orthodontic extrusion. Secondly, removable appliances placed on mucosa lead to alveolar bone resorption [44]. Due to the age of the patient and the initial bone insufficiency, the prosthetic rehabilitation was postponed until the end of the orthodontic treatment. The patient planned fixed prosthetic restorations with the use of dental implants. 

The display of the teeth was initially compromised. The patient showed less than 75% height of the central incisors, and the smile arch was distorted. The gingival display was accurate after finishing the orthodontic treatment. The midlines were correlated, and the incisal edges of the teeth were parallel to the curvature of the lower lip. Minimal overbite was present. The buccal corridors remained because the complete restorations were not performed yet. The microesthetics were distorted before the treatment, the height–width relationships of each tooth were not accurate, and the teeth were too short in relation to their width [45]. The patient decided on E-max veneers to achieve better esthetics with low-invasive preparation of the teeth. Improvement of gingival height, shape, contour, and connectors was achieved. 

The fixed upper and lower retainers were performed as a part of the regular retention phase to avoid any alterations in the positions of the teeth. The fixed upper retention was also highly indicated because the patient had diastema before the treatment. There was no pull syndrome and no invagination of the soft tissue after the diastema closure. Therefore, no frenectomy was performed. 

The presence of the impacted teeth prolonged the treatment. The PDCs needed to be moved long distances and to be properly aligned. The usage of the MIs shortened the treatment period with fixed straight-wire appliances by 8 months (active traction time with MIs only). Consequently, the risk of complications of decalcification, dental caries, and root resorption decreased. On the other hand, the treatment time was extended due to the worldwide COVID-19 pandemic and the resultant limited access to orthodontic care in our division. The patient was forced to wait over 6 months without orthodontic control during this period.

According to the patient’s opinion about the treatment, the visit for MI insertion and cantilever placement was the most uncomfortable. It was tiring due to the long duration of the appointment. The pain during the treatment was reported as minimal to none. The most difficult part of the whole process was eating with the cantilevers in place in the oral cavity. Moreover, the fracture of the cantilever was uncomfortable due to the soft tissue irritation. The patient expresses no fear of any further medical procedures to be performed during the restoration phase.

## 5. Conclusions

The use of MIs for the extrusion od PDCs makes it possible to offer this treatment option to patients with oligodontia, which could not be treated in this way previously. The presented protocol was effective and served to overcome treatment limitations associated with the inadequate amount of dental anchorage and the high risk of complications. CBCT is a useful tool for orthodontic diagnostic purposes, MI position planning, and assessment of the complications of treatment.

## Figures and Tables

**Table 1:** Cephalometric analysis.

Measurement	Norm	Deviation	Value
SNA	82.0°	± 3.0	82.9°
SNB	80.0°	± 3.0	83.6°
ANB	2.0°	± 2.0	−0.7°
SNPg	81.0°	± 3.0	86.0°
GntgoAR	122.0°	± 7.0	121.8°
NL-NSL	8.0°	± 4.0	3.1°
ML-NSL	28.0°	± 5.0	26.2°
ML-NL	20.0°	± 7.0	23.1°
1+:NA	21.0°	± 4.0	17.2°
1+:NA (mm)	3.7 mm	± 2.0	3.2 mm
1+:NL	110°	± 6.0	104°
1−:NB	24.0°	± 4.0	10.5°
1−:NB (mm)	3.8 mm	± 2.0	0.4 mm
1−:ML	94°	± 7.0	79.2°
1−:APg	1.0 mm	± 2.0	−1.4 mm
1+:1−	133.0°	± 8.0	153.5°
UL—“E” plane	−4.7 mm	± 2.0	−4.35 mm
LL—“E” plane	−2.0 mm	± 2.0	−4.45 mm

**Table 2:** Treatment process.

Treatment Period	Treatment Procedures
October 2017	PDCs’ surgical exposure—open technique; brackets bonded on PDCs; MIs tomas^®^-pin SD 06 (Dentaurum, Ispringen, Germany) inserted in the palatal alveolar region; 0.016″ × 0.022″ TMA cantilevers activated in distal and downward direction (50 g) (Figure 6a,b).
November 2017	Visible orthodontic movement—exclusion of primary ankylosis (Figure 6c).
December 2017	Increased mobility and tenderness of PDCs, dismantlement of cantilevers, button on the buccal side of UL3 (derotation), power chains to move the teeth (Figure 6d).
January 2018	Teeth stability improved; 0.017″ × 0.025″ TMA cantilever with activation in downward and buccal directions for UR3, power chain for distal movement and derotation of UL3.
February 2018	Button on the buccal side of UR3 (derotation), power chains for distal movements and derotations (Figure 6e).
April 2018	Extraction of the upper right deciduous canine due to the collision with movement of UR3; 0.017″ × 0.025″ TMA cantilever activated for buccal movement of UR3 (50 g) (Figure 6f).
June 2018	Partial fixed upper SS appliance Equilibrium^®^ 2 0.022″ in Roth prescription (Dentaurum, Ispringen, Germany), 0.016″ NiTi wire, continuous metal ligature to create space for UL3; cantilever activation (Figure 6g).
August 2018	Bracket on tooth no. 24; 0.016″ NiTi wire and open coil spring to create space for UL3; extraction of the upper left deciduous canine.
October 2018	New MI tomas^®^-pin SD 08 (Dentaurum, Ispringen, Germany) was inserted in the buccal surface of left alveolar ridge; 0.017″ × 0.025″ TMA cantilever for buccal movement of UL3 (50 g) (Figure 6h).
November 2018	Mobility of the buccal alveolar MI, the miniscrew was tightened and left to stabilize for a month; tooth no. 55 was extracted due to progressive reinclusion.
December 2018	Buccal alveolar MI was lost and new MI tomas^®^-pin SD 10 (Dentaurum, Ispringen, Germany) was placed in the IZC; 0.017″ × 0.025″ TMA cantilever with buccal activation for UL3 (50 g).
February 2019	Inflammation and submucous abscess in the IZC; MI removal; antibiotic.
April 2019	New MI tomas^®^-pin SD 10 (Dentaurum, Ispringen, Germany) was inserted in the palatal suture and used as a direct anchorage with 0.017″ × 0.025″ SS cantilever and power chain for buccal movement of UL3 (50 g); 0.016” SS wire and bend-out for UR3 (Figure 6i).
May 2019	Overcorrection of UR3 transversal relationship, 0.018″ SS wire and bend-out for tooth no. 22, new power chain from cantilever to UL3 for its buccal movement.
July 2019	Tooth no. 22 in correct sagittal relationship; MI in the palatal suture used as an indirect anchorage: 0.017″ × 0.025 SS connection wire with tooth no. 24, 0.017″ × 0.025″ cantilever with buccal activation for UL3 (50g); 0.017″ × 0.025″ Cooper NiTi wire (Figure 6j,k).
March 2020	The correct position of UL3; tooth no. 24 showed significant mobility; control panoramic X-ray: root resorption of tooth no. 24 (Figure 7); no possibility to conduct control visits on a regular basis due to COVID-19 pandemic—next appointment took place in November 2020.
November 2020	0.019″ × 0.025″ SS wire, torque expression, closure of spaces with power chain.
March 2021	Removal of MIs, 0.021″ × 0.025″ TMA wire for torque expression in the upper arch; Fixed lower SS appliance Dentaurum Equilibrium^®^ 2 0.022″ in Roth prescription (Dentaurum, Ispringen, Germany); 0.016″ NiTi wire; elastics 4 ½ oz. from palatal buttons on teeth no. 12 and 22 to the lower arch to correct the anterior crossbite (Figure 6l).
April–June 2021	Further alignment of the lower teeth by means 0.017″ × 0.025″ NiTi, and next 0.019″ × 0.025″ SS; intermaxillary elastics 4 ½ oz and offset bends on teeth no. 12 and 22 were used to correct the anterior crossbite; elastic power chains for space closure.
August 2021	Open sinus lift surgery with porcine bone-derived grafting material (The Graft™ bone substitute cancellous granules (Purgo Biologics, Seongnam, Republic of Korea) and BioCover™ resorbable collagen membrane (Purgo Biologics, Seongnam, Republic of Korea)) was performed on the right side of the maxilla.
March 2022	Two dental implants were placed: tooth no. 14—Axiom^®^ PX 3.4 × 12 mm (Anthogyr, Sallanches, France), tooth no. 15—Axiom^®^ PX 3.4 × 10 mm (Anthogyr, Sallanches, France).
May–August 2022	Finishing; control panoramic X-ray (Figure 8); 1st canine relationships and midline consistency; debonding of the brackets; fixed upper and lower retainers’ placement (0.027″ × 0.011″ 8-strand braided SS), tooth no. 24 was not fixed to the retainer due to increased mobility.
September 2022	E-max (lithium desilicated ceramic) veneers on upper teeth; individual implant abutments (titanium pre-milled abutments) and implant-supported blocked crowns (zirconia veneered with porcelain using the cut-back technique) on dental implants; removable thermoformable retainer.
March 2023	Retention phase: 10-month follow-up. Stability of treatment results; minimal opening of the spaces mesially to teeth no. 34 and 44; proper mobility of tooth no. 24. Bleaching of the lower teeth.

IZC—infrazygomatic crest area, MI—mini-implant, NiTi—nickel–titanium, no.—number, SS—stainless steel, TMA—titanium molybdenum, UL3—upper left canine, UR3—upper right canine.

**Table 3:** Changes in cephalometric measurements after treatment.

Measurement	Pretreatment	Posttreatment	Difference
SNA	82.9°	83.4°	0.5°
SNB	83.6°	85.0°	1.4°
ANB	−0.7°	−1.6°	−0.9°
SNPg	86.0°	87.7°	1.7°
GntgoAR	121.8°	116.3°	−5.5°
NL-NSL	3.1°	4.8°	1.7°
ML-NSL	26.2°	22.0°	−4.2°
ML-NL	23.1°	17.2°	−5.9°
1+:NA	17.2°	32.1°	14.9°
1+:NA (mm)	3.2 mm	5.4 mm	2.2 mm
1+:NL	104°	117.2°	13.2°
1−:NB	10.5°	13.7°	3.2°
1−:NB (mm)	0.4 mm	−0.1 mm	−0.5 mm
1−:ML	79.2°	85.7°	6.5°
1−:APg	−1.4 mm	−1.2 mm	0.2 mm
1+:1−	153.5°	135.8°	−17.7°
UL—“E” plane	−4.35 mm	−5.02 mm	−0.67 mm
LL—“E” plane	−4.45 mm	−3.62 mm	0.83 mm

**Table 4:** The lengths of the roots before and after the treatment.

Tooth Number	13	12	11	21	22	23	24
Root length before treatment (mm)	11.5 + 2.1 *	12.9	11.1	10.8	12.8	14.6	9.4
Root length after treatment (mm)	11.0	12.3	10.5	9.5	10.9	12.4	8.5

* dilaceration.

## Data Availability

The data presented in this study are available upon request from the corresponding author. The data are not publicly available due to privacy restrictions.
